# Ferroptosis and Nrf-2 signaling: a redox tug of war in leishmaniasis pathogenesis and host directed therapy

**DOI:** 10.1016/j.redox.2026.104071

**Published:** 2026-02-03

**Authors:** M. Junaid Dar, Ryan H. Huston, Nandithadas Purayil, Ashley I. Cronin, Sonia Ashfaq Gondal, Hira L. Nakhasi, Reginaldo Brito, Sreenivas Gannavaram, Abhay R. Satoskar

**Affiliations:** a Department of Microbiology, The Ohio State University, Columbus, OH, 43210, USA; b Department of Pathology, Wexner Medical Center, The Ohio State University, Columbus, OH, 43210, USA; c School of Pharmacy, University of Management and Technology, Lahore, Pakistan; d Laboratory of Emerging Pathogens, Center for Biologics Evaluation and Research, US Food and Drug Administration, 10903 New Hampshire Ave, Silver Spring, MD, 20993, USA; e Laboratório de Patologia Estrutural e Molecular (LAPEM), Instituto Gonçalo Moniz, Fundação Oswaldo Cruz, Salvador, Brazil; f Departamento de Patologia e Medicina Legal, Faculdade de Medicina, Universidade Federal da Bahia, Salvador, Brazil

**Keywords:** Nrf-2, Ferroptosis, Leishmaniasis, Redox homeostasis, Macrophages, Host directed therapy

## Abstract

Leishmaniasis is a neglected intracellular parasitic disease, where host response is shaped not only by Th1/Th2 immune polarization but also by iron availability, redox homeostasis and regulation of cell death pathways. Ferroptosis, a unique iron dependent cell death pathway has recently emerged as a critical mechanism in innate host protection against intracellular pathogens. In *Leishmania* infected macrophages, disruption of iron homeostasis and promotion of oxidative stress support an environment favorable for ferroptosis. However, parasites employ cytoprotective and antioxidant pathways, most importantly the nuclear factor erythroid 2-related factor (Nrf-2) signaling, to evade the ferroptosis mediated host response. Nrf-2 activates a wide array of antioxidant pathways, limiting ferroptosis and promoting parasites survival within macrophages. The balance between ferroptotic stress and Nrf-2 mediated antioxidant defense varies between cutaneous and visceral forms of leishmaniasis. In cutaneous leishmaniasis, early oxidative stress favors ferroptotic parasite control and transient Nrf-2 activation preserve tissue integrity, however sustained Nrf-2 activation suppresses ferroptosis and favors infection. In contrast, visceral leishmaniasis is characterized by systemic iron dysregulation and persistent Nrf-2 activation which creates a ferroptosis resistant environment. Targeting this context dependent ferroptosis/Nrf-2 axis offers a promising host directed strategy for leishmaniasis. Therapeutic approaches involving ferroptosis induction and Nrf-2 suppression may counteract ferroptosis resistance driven by antioxidant defenses and restore host antipathogen functions. This review integrates current insights into ferroptosis and Nrf-2 signaling in both cutaneous and visceral leishmaniasis. It further highlights potential therapeutic agents targeting ferroptosis and Nrf-2 pathways as promising candidates for host directed therapeutic strategies in leishmaniasis.

## Highlights

• Ferroptosis represents an iron dependent form of oxidative cell death driven by lipid peroxidation.
• Disruption of iron homeostasis promotes lipid peroxidation and ferroptotic vulnerability.
• Nrf-2 mediated induction of antioxidant, iron-sequestering and NADPH generating enzymes suppresses ferroptotic damage and promotes intracellular pathogen survival.
• The two major clinical forms of leishmaniasis exhibit distinct patterns of ferroptotic stress and Nrf-2 activity, shaping their respective disease outcome.
• Therapeutic strategies that adjust redox homeostasis and ferroptosis pathways could enhance host-directed control of leishmaniasis.

## Graphical abstract

**Figure:**
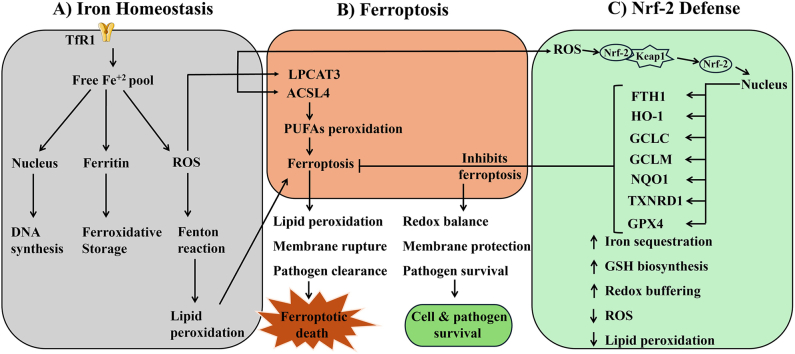

## 1 Introduction

Leishmaniasis is one of the 21 neglected tropical diseases recognized by the World Health Organization (WHO). It is caused by protozoan parasites of the genus *Leishmania*, which are transmitted through the bite of an infected female phlebotomine sandflies. The disease ranges from self-healing cutaneous lesions to a severe life-threatening visceral form. It is the second most fatal parasitic disease after malaria. Annually, 0.7-1.2 million cases of cutaneous leishmaniasis (CL) occur globally, with more than 500 million people at risk, whereas visceral leishmaniasis (VL) affects about 0.2-0.4 million people and causes nearly 20,000 deaths [1]. Leishmaniasis cases have been increasingly reported in the United States, especially in the southern states of Texas and Oklahoma. Climate change and travel are expanding the geographic range of sandfly vectors toward the north side, which may transform leishmaniasis from a an uncommon disease to an endemic threat in other US states [2].

Macrophages are the primary host cells of *Leishmania* parasites and are the center of competing parasitic metabolic manipulation and host directed cytotoxic mechanisms that determine the fate of infection. In leishmaniasis, research has primarily focused on the balance between Th1 and Th2 immunological responses and the molecular mechanisms of macrophage activation. A robust Th1 response stimulates M1 pro-inflammatory polarization of macrophages which produce nitric oxide (NO) and reactive oxygen species (ROS) to kill intracellular *Leishmania* parasites. On the other hand, a strong Th2 response promotes M2 anti-inflammatory polarization and encourages IL-4 and IL-10 secretion which supports parasite survival and chronic infection [3]. However, in recent years, emerging work on regulated cell death pathways and macrophage immunometabolism revealed that the outcome of infection is not merely shaped by Th1/Th2 polarization but also by the metabolic state and death susceptibility of host cells.

Apoptosis, necrosis and pyroptosis cell death pathways have been extensively studied in infectious diseases but a non-apoptotic and iron dependent cell death pathway, known as ferroptosis, has recently surfaced as an important determinant of host-pathogen dynamics. Ferroptosis is characterized by the accumulation of reactive lipid peroxides in the cells, resulting from iron overload which causes membrane damage and cell death, and is distinct from other cell death mechanisms. Insights into ferroptosis provide a unique framework to understand iron homeostasis, oxidative stress and lipid peroxidation interconnections in infectious intracellular diseases, such as leishmaniasis that exploit host metabolic pathways [4]. Recent studies suggest that ferroptosis or the host's ability to undergo ferroptotic death is the main mechanism that could decide the fate of parasitic infection [5,6]. The parasites create a protective intracellular microenvironment by sequestering enough iron for their own DNA synthesis and replication while modifying the host antioxidant defense system in their favor to downregulate host iron toxicity responses. Stimulation of ferroptosis within *Leishmania* infected macrophages may limit parasite replication by enhancing intracellular iron toxicity and disrupting the antioxidant defense system on which parasites rely for survival.

There has been a growing focus on interpreting host antioxidant mechanisms that could decrease ferroptotic stress. Among these, the Nuclear factor erythroid 2-related factor 2 (Nrf-2) pathway has gained particular importance because of its ability to regulate iron metabolism and to suppress lipid peroxidation, which influence whether *Leishmania* infected macrophages undergo ferrototic death or not. Nrf-2 is one of the central antioxidant regulatory pathways that links redox control and cell death susceptibility. It regulates several genes that control iron storage, detoxify ROS and restore redox equilibrium. In normal conditions, Nrf-2 is confined in the cytoplasm binding with Kelch like ECH associated protein 1 (Keap1) and is marked for ubiquitin mediated degradation, where small ubiquitin molecules are attached to Nrf-2, targeting it for proteasomal breakdown. When cells are exposed to oxidative stress, Nrf-2 dissociates from Keap1, translocates to the nucleus and activates transcription of several important genes that encode antioxidant and cytoprotective proteins such as glutathione peroxidase 4 (GPX4), heme oxygenase-1 (HO-1), cystine/glutamate antiporter system x_c_^−^, solute carrier family 7 member 11 (SLC7A11) and many others [7]. Although Nrf-2 activation is important for protecting normal cells under oxidative stress, its upregulation in *Leishmania* infected macrophages support parasite survival by reducing oxidative damage and preventing ferroptotic death [6,8]. In short, while *Leishmania* depends on host derived iron to sustain its metabolic needs, its ability to activate the Nrf-2 pathway enables the parasite to neutralize oxidative stress and evade ferroptosis and promotes its intracellular persistence.

In leishmaniasis, investigating the roles of ferroptosis and Nrf-2 may open new frontiers in the research field of anti-leishmanial drug development. Ferroptosis is a component of innate immune defense that helps to eliminate parasites and Nrf-2 suppress this defense. Exploration of these pathways could provide valuable insights and offer a novel therapeutic strategy to eradicate parasites that have evolved to escape these responses.

## 2 Ferroptosis

Ferroptosis is a distinct form of cell death, characterized by the peroxidation of polyunsaturated fatty acids (PUFAs), a process mediated by intracellular iron dependent oxidative reactions. It differs from apoptosis which involves caspase activation and DNA fragmentation or necrosis which is unregulated and depicted by loss of cellular membrane integrity. Ferroptosis was first reported in 2012 and since then it has been studied as a critical process in various diseases such as cancer, neurodegeneration, injury and infectious diseases [9,10]. Ferroptosis has recently gained attention as a regulated cell death mechanism in leishmaniasis, characterized by dysregulated iron homeostasis and oxidative stress, both of which are hallmark features of *Leishmania* infected macrophages [6].

Ferroptosis emerges as a result of dysregulation of three interrelated processes: 1) iron metabolism 2) lipid peroxidation and 3) the antioxidant defense system. Iron is imported into the cytosol and performs several biochemical reactions, such as DNA synthesis, mitochondrial respiration and oxygen transport, but excessive free iron can catalyze the Fenton reaction, converting hydrogen peroxide into highly reactive hydroxyl radicals, resulting in the oxidation of lipids, nucleic acids and proteins, which may lead to ferroptotic cell death. Iron homeostasis in cells is regulated through the coordinated teamwork of transferrin receptor 1 (TfR1), divalent metal transport 1 (DMT1), ferritin, ferroportin and iron regulatory proteins (IRPs). In normal healthy cells, TfR1 facilitates the cellular uptake of transferrin bound iron (Fe^3+^), which circulates in the blood bound to transferrin to prevent free iron induced oxidative damage. Inside the cell, Fe^3+^ is released in its free ferrous form (Fe^2+^) and transported into the cytosol by DMT1. Ferritin is an iron storage complex, binding excess free iron and preventing its involvement in the Fenton chemistry. On the other hand, iron levels that exceed cellular needs are exported out of the cell through ferroportin and this network is regulated by IRPs, which are regulatory proteins that sense intracellular iron levels and control the expression of iron modulating genes to maintain iron homeostasis [11,12]. However, disruption of this intracellular iron homeostasis leads to iron dependent oxidative stress and subject cells to ferroptosis. The excessive free iron causes the oxidation of PUFAs, which are embedded within phospholipids of cellular membranes and creates substrates that are vulnerable to peroxidation. It initiates chain reactions which results in compromised membrane integrity and ferroptotic cell death. Cells employ an antioxidant defense system to counter this oxidative stress using the GPX4 enzyme that reduces lipid hydroperoxides to their corresponding alcohols and halts the process of peroxidation. GPX4 action is dependent on the availability of glutathione (GSH) and the import of cystine through the cystine/glutamate antiporter system xc^−^, which are limiting steps in the synthesis of GSH [13].

Several mediators interconnect metabolic and signaling pathways within the ferroptotic program, including p53 (a tumor suppressor that plays a role in apoptosis), which can also modulate ferroptosis by suppressing SLC7A11 transcription, leading to cystine starvation. p53 promotes oxidative stress, apoptosis and ferroptosis under these conditions. On the other hand, Nrf-2 pathway suppresses ferroptosis by upregulation of antioxidant and iron binding genes, including ferritin heavy chain (FTH1), HO-1 and NADPH generating enzymes [14]. The regulatory tug of war between p53 and Nrf-2 decides whether a cell undergoes oxidative elimination or survives, a mechanism exploited by several intracellular pathogens to facilitate their survival within host cells.

### 2.1 Ferroptosis in parasitic pathogenesis

The availability of iron is one of the most important factors that decides the outcome of a parasitic disease, as it is involved in host and parasite DNA replication, oxidative metabolism and many other functions, but excessive free iron contributes to oxidative stress and drives ferroptotic lipid peroxidation in host cells. However, parasites have evolved to exploit host iron metabolism in their favor while minimizing oxidative toxicity. The role of ferroptosis in parasitic diseases has emerged from several studies in malaria and trypanosomiasis. *In* *vitro* and *in vivo* animal models, it was found that exposure to ferroptosis inducing drugs such as sorafenib or erastin resulted in a reduction of *Plasmodium* infection, whereas the use of ferrostatin (an anti-ferroptotic agent) increased parasitic burden [15]. This study offered evidence that the ferroptosis mechanism is important for host defense against intracellular parasites. Another study reported that dihydroartemisinin (DHA), an established antimalarial drug, displayed therapeutic results against *Toxoplasma gondii* in *in* *vitro* (cell culture) studies by increasing parasite induced ROS production and disrupting mitochondrial membrane potential. However, these changes were reversible with ferroptosis inhibitors or iron chelators, suggesting role of ferroptosis in disease pathogenesis. RSL3, a well-characterized ferroptosis inducer, was shown to inhibit parasite growth and its co-administration with DHA resulted in enhanced antiparasitic activity [16]. Comparable findings have been reported in malaria in *in* *vitro* and *in vivo* studies, where ferroptosis inducing compounds such as RSL3, Erastin and Sorafenib exhibited inherent antiparasitic activity. Their effects were strengthened when combined with DHA, whereas ferroptosis inhibitors like deferoxamine and liproxstatin-1 reduced DHA's efficacy. These observations suggest that ferroptosis related pathways contribute to the antiparasitic action of DHA [17]. These findings indicate that inducing ferroptosis can impair parasites survival within cells and position ferroptosis as a promising therapeutic target for antiparasitic drug discovery.

### 2.2 Ferroptosis in leishmaniasis

In *Leishmania* pathogenesis, ferroptosis is linked to host iron metabolism, redox regulation and immune polarization. The different metabolic and immune landscapes of CL and VL offer a unique platform to study how these factors shape ferroptotic susceptibility in *Leishmania* infected macrophages. Several factors including macrophage subpopulations, inflammatory cytokines release and organ specific environments could determine whether *Leishmania* infected macrophages shift toward a ferroptosis permissive or ferroptosis resistant state. The study of manipulation of iron homeostasis, lipid metabolism and antioxidant defenses in leishmaniasis to suppress ferroptotic death may reveal innovative host directed strategies to control the infection. Ferroptosis is a recently explored form of cell death, thereby, only a few studies have explored its role in leishmaniasis.

#### 2.2.1 Ferroptosis and cutaneous leishmaniasis

CL is caused by several species of *Leishmania* including *L. major*, *L. tropica* and *L. mexicana*, and manifests as localized skin lesions. In CL, macrophages, neutrophils and dendritic cells coordinate the host immune response. The prevalent Th1 immune response stimulates the production of TNF-α and IFN-γ, that activates macrophages and their polarization towards M1 phenotype. M1 macrophages then metabolically reprogram toward glycolysis and result in the generation of ROS and NO, thereby developing an oxidative environment detrimental to intracellular parasites. Moreover, the activation of macrophages, during early IFN-γ signaling, can increase free iron availability and promote lipid peroxidation derived by ROS [3,18]. Also, transcriptomic studies of *Leishmania* infected macrophages revealed that there were upregulation of lipid remodeling and pro-oxidant genes [19,20], associated with ferroptosis related pathways. At the same time, *Leishmania* in early infection responds to host oxidative defenses through an increase in surface glycoconjugates such as lipophosphoglycan (LPG) that inhibits NADPH oxidase 2 (NOX2) dependent ROS production and activation of Nrf-2 mediated HO-1 antioxidant system. It decreases free iron availability and detoxify lipid hydroperoxides to prevent ferroptosis [21,22]. These regulations suggest that CL infection resides in a metabolic grey area, where iron acquisition and antioxidant responses are balanced in such an efficient way that it maintains a favorable environment for parasite survival. Therapeutic modification of this balance through increase in free iron, inhibition of antioxidant defenses and stimulation of lipid peroxidation, could unmask the susceptibility of infected macrophages to ferroptosis and offers a novel host directed strategy against CL.

Several *in vivo* studies highlight the modulation of iron homeostasis in CL and how it intersects with redox dependent antiparasitic defenses. In a study, the alteration of systemic iron levels in BALB/c mice switched disease severity following *L. major* infection. Iron supplementation reduced lesion development and significantly reduced parasite burden in the skin and draining lymph nodes. This effect was accompanied by decreased IL-4/IL-10 and increase IFN-γ/iNOS transcription at lesion sites, indicating a shift towards a protective Th1 immune response [23]. Subsequent work established that iron supplementation in mice exhibited a significantly enhanced oxidative burst and this ROS driven response plays an important role in parasite control, however, antioxidant treatment reversed this protective phenotype. Importantly, in low iron dose challenge models, sustained oxidative activity in the ear dermis prevented lesion formation for over a year and demonstrates that iron induced redox pressure can create long term resistance to parasites [24]. It was also revealed in BALB/c mouse models that iron mediated protection was associated with increased T cell proliferation, enhanced NF-κB activation and elevated IFN-γ producing CD4^+^ T cells [25]. This suggests that systemic iron not only amplifies early innate oxidative mechanisms but also enhances the development and maintenance of adaptive antileishmanial immunity.

#### 2.2.2 Ferroptosis and visceral leishmaniasis

VL is caused by *L. donovani* and *L. infantum*, and differs from CL in its systemic pathology. In VL, unlike CL where local oxidative stress prevails, parasites spread throughout the spleen, liver and bone marrow, and results in widespread immune suppression and iron homeostasis disruption. The liver and spleen are major pathological sites of VL and propose valuable perspectives into ferroptotic dynamics. In both experimental and clinical VL, histopathological analysis of the liver and spleen in general displays widespread macrophage infiltration and disruption of tissue physiology but unexpectedly limited necrotic cell death [26], indicating that cell death pathways are not dominant in these lesions. Although ferroptosis has not yet been comprehensively evaluated as a cell death pathway in VL, the lack of extensive tissue damage typically associated with oxidative stress raises the possibility that ferroptotic mechanisms are constrained or counterbalanced during infection.

Chronic inflammation in VL patients is associated with an increase in hepcidin expression, which binds to ferroportin and triggers its degradation. This reduces iron export from macrophages, trapping iron inside the cell and reducing serum iron availability [27]. In parallel, parasites present within host cells interfere with intracellular iron storage by cleaving chaperone proteins that load iron into ferritin. In murine macrophage cell lines (J774) and mouse splenocytes, this disruption increases the pool of free iron and is exploited by parasites for their DNA synthesis and replication [28]. However, in mouse models and human macrophages, lipid peroxidation remains suppressed due to elevated ferritin and HO-1 expression which creates iron rich, but ROS protected intracellular environment [29]. This shows that although VL creates conditions of iron accumulation, parasites activate strong anti-oxidant countermeasures that suppress ferroptotic stress.

Experimental data show that excessive iron availability can overcome the oxidative protective mechanisms and significantly reduce parasite burden in the liver and spleen. This inhibitory effect depends on host NADPH oxidase and NO synthase (NOS) activity, as iron synergizes with reactive iron and nitrogen species to overcome parasite defenses. The effect was absent in oxidase or NOS deficient mice, confirming that iron toxicity works through host oxidative pathways rather than adaptive immunity [30]. In another study it is reported that liver resident Kupffer cells display ferroptotic cell death, characterized by iron dependent oxidative damage and lipid peroxidation, in a chronic murine VL experimental model. This process results in clearance of infected macrophages and is associated with reduced intracellular parasite survival [31]. Iron overload appears to induce oxidative stress beyond the antioxidant capacity and share conceptual similarities with ferroptotic pathways. Taken together, these observations indicate that suppression of ferroptosis and upregulation of antioxidant pathways such as, Nrf-2, are successful strategies adopted by *Leishmania* parasites to establish chronic VL infection.

## 3 Crosstalk between ferroptosis and Nrf-2

**Fig. 1:**
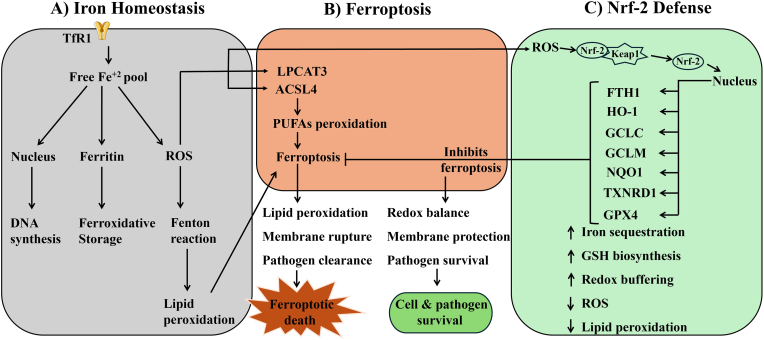
Interplay between iron metabolism, ferroptotic pathways and Nrf-2 driven antioxidant defense in macrophages during *Leishmania* infection. (A) Iron regulation: Transferrin receptor 1 (TfR1) facilitates uptake of transferrin-bound ferric iron (Fe^3+^) which is subsequently reduced to ferrous iron (Fe^2+^) inside the cell. This free iron pool contributes to nuclear DNA synthesis, is stored in ferritin, or participates in Fenton chemistry, generating reactive oxygen species (ROS). Elevated ROS promotes lipid peroxidation of polyunsaturated fatty acids (PUFAs), initiating ferroptotic stress. (B) Ferroptosis: Disrupted iron balance and oxidative pressure activate lipid remodeling enzymes such as lysophosphatidylcholine acyltransferase 3 (LPCAT3) and acyl-CoA synthetase long-chain family member 4 (ACSL4), driving PUFA peroxidation and membrane destabilization. This results in ferroptotic cell death, which can aid pathogen clearance. (C) Nrf-2 mediated defense: Oxidative signals modify Kelch-like ECH-associated protein 1 (Keap1), releasing nuclear factor erythroid 2-related factor 2 (Nrf-2) to translocate into the nucleus and induce antioxidant response element (ARE) regulated genes. These include ferritin heavy chain (FTH1), heme oxygenase-1 (HO-1), glutamate-cysteine ligase catalytic (GCLC) and modifier (GCLM) subunits, NAD(P)H quinone oxidoreductase 1 (NQO1), thioredoxin reductase 1 (TXNRD1) and glutathione peroxidase 4 (GPX4). Collectively, these pathways promote iron sequestration, glutathione (GSH) synthesis and redox buffering, thereby limiting lipid peroxidation, suppressing ferroptosis and supporting parasite survival.

The Nrf-2 pathway is one of the oxidative defense mechanisms that a cell employs to overcome oxidative challenge and is important for cellular survival. It was discovered as a member of the cap ‘n’ collar family of transcription factors and central regulator of oxidative burden, cytoprotection and metabolic reprogramming. Although Nrf-2 activation is crucial for maintaining cell viability under redox imbalance but in several pathological conditions, including cancer, chronic inflammation or infection, its persistent or dysregulated activation promotes survival of infected cells and contributes to disease progression. The interplay between iron metabolism, ferroptotic pathways and Nrf-2 mediated antioxidant defense is summarized in Fig. 1.

Nrf-2 regulates more than 200 genes that are involved in several important cellular functions such as iron metabolism, redox balance, NADPH regeneration, glutathione biosynthesis and xenobiotic metabolism. One of these genes is HO-1 which catalyzes the degradation of heme into biliverdin, free iron and carbon monoxide (CO). Biliverdin and CO shows antioxidant and anti-inflammatory properties. Free iron release could promote ferroptosis through Fenton chemistry, however, simultaneous upregulation of ferritin and GPX neutralizes this stress [32]. These changes maintain redox stability and enable parasite survival. Nrf-2 also regulates expression of several modifier subunits such as glutamate cysteine ligase catalytic and modifier subunits (GCLC and GCLM) which are involved in the synthesis of GSH that is a cofactor for GPX4, the principal intracellular antioxidant enzyme which prevents lipid peroxidation in ferroptosis. Similarly, Nrf-2 promotes NAD(P)H quinone oxidoreductase 1 (NQO1) and thioredoxin reductase 1 (TXNRD1), which are important enzymes that support the cellular redox buffer. NQO1 promotes the reduction of quinones to hydrquinones, blocking their conversion into semiquinone radicals (which accelerates ROS and promotes lipid peroxidation). TXNRD1 keeps TXN in its reduced state and enables the regeneration of thiols and limits oxidative protein damage. If these thiols are not reduced, oxidized proteins would accumulate and the cellular redox balance shifts in a way that promotes lipid peroxidation and ferroptosis [33]. Nrf-2 activation also enhances the expression of ferritin heavy and light chain expression, involved in sequestering surplus cytosolic iron, and inhibits the expression of DMT-1 and ferroportin, overall shifting the homeostasis toward iron storage and reducing iron flux. In addition, Nrf-2 improves the expression of SLC7A11 (a subunit of the system x_c_^−^ antiporter) responsible for cysteine uptake and maintaining cysteine availability for GSH synthesis [34,35].

Collectively, these mechanisms allow Nrf-2 to suppress ferroptosis. Nrf-2 directly promotes GPX4 activity through preserved GSH levels and restricting the pool of reactive iron required to drive lipid peroxidation. Meanwhile, Nrf-2 indirectly affects ferroptosis by reducing ROS generation and limiting the overall oxidative burden.

### 3.1 Ferroptosis and Nrf-2 interplay in leishmaniasis

The crosstalk between ferroptotic and Nrf-2 pathways forms a key nexus that decides the fate of infected macrophages survival and thereby determining the outcome of chronic *Leishmania* infection. As mentioned earlier, Nrf-2 acts as the central transcription factor regulating antioxidant protection through modulation of genes that are responsible for detoxification of lipid peroxidase, glutathione production and maintenance of iron balance, which clearly offset the execution phase of ferroptosis. This shows that the interaction of Nrf-2 with ferroptosis related pathways in *Leishmania* infected macrophages is not accidental but central to the host-*Leishmania* conflict.

Several studies reported the ferroptotic resistance role of Nrf2-GPX4 axis in the outcome of infection by *Leishmania* parasites. In a recent study, primary macrophages obtained from mice infected with *Leishmania* showed Nrf-2 activation, suppress ferroptotic death program and enabled parasite survival [6]. Similarly, in an *in vivo* murine model, T cells lacking GPX4 accumulate membrane lipid peroxides and undergo ferroptotic cell death. These T cells fail to expand and mediate protective immunity upon infection [36]. Recent evidence also suggests that during early *Leishmania* infection NOX2-ROS is activated in the macrophages and it does not function as cytotoxic agents but as a signaling molecule that oxidizes Keap1 and initiates Nrf-2 activation. This early redox signal is further strengthened by prostaglandin E2 (PGE2)-EP2 receptor signaling, a pathway reported to promote cAMP driven anti-inflammatory programs and sustain Nrf-2 activity. The activation of Nrf-2 through several mechanism create an antioxidant environment that protects infected macrophages from ferrotptotic death [6]. *Leishmania* infected macrophages encounter a burst of reactive oxygen and nitrogen species generated through inducible nitric oxide synthase (iNOS) and NADPH oxidase, to eliminate intracellular pathogen. However, evidence suggests that *Leishmania* exploit these oxidative defenses to their survival by triggering Nrf-2 activation and its downstream antioxidant pathways [37].

A growing body of evidence suggests that Nrf-2 activation during *Leishmania* infection is not an ordinary bystander response, but rather an actively regulated pathway hijacked by the pathogen. In *Leishmania* infection, the activation of Nrf-2 is linked to autophagy dependent degradation of Keap1 and an increase in expression of the autophagy adaptor LC3-II conversion, p62/SQSTM1 and formation of a p62-Keap1 complex, which results in Keap1 degradation and the release of Nrf-2 for nuclear translocation [38]. Proteomic profiling has provided further insights into the interaction between ferroptotic and antioxidant pathways during *Leishmania* infection. Proteomic analysis of bone marrow derived macrophages from CBA mice, upon infection with *L. amazonensis*, demonstrated a robust activation of Nrf-2 regulated antioxidant response, characterized by upregulation of HO-1 and sequestosome-1 (p62) with enhanced uptake of holo-transferrin uptake into parasite loaded vacuoles in the macrophages [39].

The crosstalk of ferroptosis and Nrf-2 is not limited to redox homeostasis but also triggers metabolic and immunological shifts. As discussed earlier, alteration in lipid metabolism is observed in macrophages undergoing ferroptosis stress which favors accumulation of PUFA in host membrane phospholipids and results in increase in susceptibility to lipid peroxidation. Nrf-2 controls the expression of lipid modifying enzymes such as ACSL3 and lysophosphatidylcholine acyltransferase, and adjusts the membrane lipid profile, which helps determine the ferroptotic vulnerability of the host cells. The activation of Nrf-2 triggers metabolic rewiring in macrophages and stimulates fatty acid oxidation and mitochondrial biogenesis which in turn results in decreasing PUFA hydroperoxides accumulation in the host cell and ensures cell viability [40].

A study suggests that Nrf-2 activation promotes an environment that shifts macrophages from M1 to M2 phenotype [41], that supports parasites survival and prevents infected macrophages from ferroptotic death. Leishmaniasis promotes intracellular calcium fluxes and mitochondrial disruption that boosts ROS production, sustaining Nrf-2 activation in a positive feedback mechanism. Interestingly, this persistent Nrf-2 activation promotes macrophage polarization toward M2 phenotype which further weakens the pro-ferroptotic environment associated with M1 polarized macrophages. Together with these Nrf-2 mediated responses, NO synthesis was also reduced which reflects suppression of classical M1 macrophages and a phenotype shift towards M2 macrophages [6,32,38]. During chronic infection, several anti-inflammatory cytokines including IL-10 and TGF-β are released by infected macrophages and it is reported that these promote antioxidant and cytoprotective pathways which in turn favors Nrf-2 activation and supports an anti-ferroptotic environment favorable to *Leishmania* persistence [6]. This indicates that *Leishmania* induced Nrf-2 activation not only prevents premature host cell death that would eliminate parasites habitat but at the same time suppresses immune effector responses to promote intracellular parasites survival. During *Leishmania* pathogenesis, the functional roles of ferroptosis related molecules, their relation to ferroptotic death and regulation by Nrf-2 signaling pathway are summarized in Table 1.

**Table 1:** Functional roles of ferroptosis related molecules, their involvement in ferroptotic cell death and regulation by the Nrf-2 signaling pathway during *Leishmania* pathogenesis.

Key molecules	Cellular function	Role in ferroptosis	Regulation by Nrf-2	Role during *Leishmania* infection
TfR1, DMT	Iron uptake	Promotes ferroptosis by increasing free Fe^2+^ pool	Indirect suppression via ferritin induction	Iron influx supports replication, while Nrf-2 activation counters ROS
Ferritin	Iron storage	Iron sequestration and reduce lipid peroxidation.	Indirect suppression	Iron-rich but ferroptosis resistant environment
Ferroportin	Iron export	Removes excess intracellular iron	Suppressed via hepcidin–Nrf-2 axis	Promotes iron efflux and limits ferroptotic death
ACSL4, LPCAT3	Lipid metabolism	PUFA incorporation triggers ferroptosis.	Nrf-2 limits PUFA remodeling	Ferroptotic susceptibility is reduced
ROS	Lipid peroxidation	Enhances ferroptosis through membrane oxidation.	Neutralized by antioxidant enzymes.	Parasite survival favored
GPX4, GSH	Antioxidant defense	Detoxifies lipid hydroperoxides and suppress ferroptosis	Strongly upregulated	Blocks ferroptosis execution
System x_c_^−^ (SLC7A11)	Cystine uptake and GSH synthesis	Sustains GSH synthesis and suppress ferroptosis	Direct transcriptional target	Maintains redox homeostasis
HO-1	Heme metabolism	Releases iron but coupled with ferritin induction	Nrf-2 target gene	Net anti-ferroptotic effect
NQO1, TXNRD1	Redox buffering	Maintains NADPH and thiol pools	Strongly induced	Suppresses oxidative stress

ACSL4: Acyl CoA synthetase long chain 4, DMT: Divalent metal transporter, GPX4: Glutathione peroxidase 4, GSH: Glutathione, HO-1: Heme oxygenase 1, LPCAT3: Lysophosphatidylcholine acyltransferase 3, NQO1: NAD(P)H quinone dehydrogenase 1, Nrf-2: Nuclear factor erythroid 2 related factor, PUFA: Polyunsaturated fatty acids, ROS: Reactive oxygen species, TfR1: Transferrin receptor 1, TXNRD1: Thioredoxin reductase 1.

Taken together, the ferroptosis-Nrf-2 network emerges as a flexible and complex system within *Leishmania* infected macrophages, where the host and parasites are in continuous biochemical tug of war for redox dominance. Understanding the delicate balance between iron homeostasis, ferroptosis and Nrf-2 could not only provide mechanistic insights into the leishmaniasis pathogenesis but also identifies a promising direction for drug development with multiple mechanistic benefits for *Leishmania* control.

### 3.2 Nrf-2 Signaling in cutaneous and visceral leishmaniasis

Although Nrf-2 is activated across various forms of leishmaniasis, its outcome depends on the tissue environment and form of leishmaniasis. In CL, Nrf-2 plays an important role in the early phase by countering the strong oxidative and inflammatory stress environment created during *Leishmania*-host interaction. This early antioxidant response is predominantly important for macrophage viability and allows these cells to withstand controlled inflammatory signaling and mechanisms that limit parasite survival. In addition, Nrf-2 performs another important function in early CL is that it restricts excessive tissue damage and limits oxidative injury to fibroblasts, keratinocytes, and surrounding skin structures. Nrf-2 activation in early CL ensures the preservation of structural integrity vital for wound healing. However, sustained Nrf-2 activation can shift from protective to pathogenic environment in chronic CL. Evidence from skin tissue samples of CL patients showed that elevated Nrf-2 and HO-1 levels in chronic CL may suppress ferroptotic cell death and allow parasite survival [42].

**Fig. 2:**
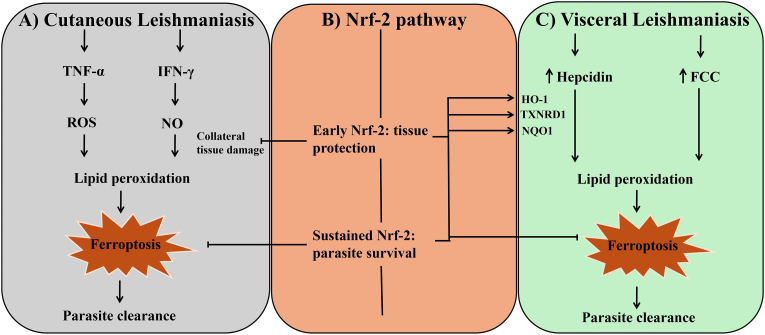
Differential regulation of ferroptosis and Nrf-2 signaling in cutaneous and visceral leishmaniasis. (A) Cutaneous leishmaniasis (CL): A strong Th1 driven immune response elevates tumor necrosis factor-α (TNF-α) and interferon-γ (IFN-γ), which activates macrophages and promotes reactive oxygen species (ROS) and nitric oxide (NO) production. This oxidative burst triggers lipid peroxidation, leading to ferroptosis and parasite clearance. However, excessive ROS and NO can cause collateral tissue injury. Early activation of nuclear factor erythroid 2-related factor 2 (Nrf-2) helps maintain tissue integrity by limiting oxidative damage, whereas prolonged Nrf-2 activity suppresses ferroptosis and favors parasite persistence. (B) Visceral leishmaniasis (VL): Systemic infection induces hepcidin expression and ferritin chaperone cleavage (FCC), disrupting iron homeostasis and increasing intracellular iron availability. Despite iron overload, elevated antioxidant defenses driven by Nrf-2 and its downstream targets such as heme oxygenase-1 (HO-1), thioredoxin reductase 1 (TXNRD1) and NAD(P)H quinone oxidoreductase 1 (NQO1), reduce lipid peroxidation and inhibit ferroptosis, enabling parasite survival.

In contrast to CL, where Nrf-2 activation is initially beneficial for maintaining macrophage viability and limiting excessive tissue injury, VL displays a different pattern in which the parasites actively exploit Nrf-2 to promote its intracellular persistence. Fig. 2 illustrates the contrasting roles of ferroptosis and Nrf-2 signaling in cutaneous versus visceral leishmaniasis. *L. donovani* infection in BALB/c mice significantly upregulates Nrf-2 which leads to increase in expression of its downstream antioxidant enzyme HO-1. This results in suppression of oxidative burst and reduction of proinflammatory cytokines, both of which are essential mechanisms for parasite control. Notably, silencing Nrf-2 not only reduces HO-1 levels but also significantly decreases parasite survival which highlights its central regulatory role in VL [43].

Taken together, these observations demonstrate that Nrf-2 functions as a context dependent regulator in leishmaniasis, where its protective antioxidant functions in CL infection contrast significantly with its parasite driven and immunosuppressive activity in VL. Table 2 summarizes the key differences in ferroptosis and Nrf-2 pathway activities between CL and VL.

**Table 2:** Ferroptosis and Nrf-2 pathway variations in cutaneous versus visceral leishmaniasis.

Specifications	Cutaneous leishmaniasis (CL)	Visceral leishmaniasis (VL)
Disease distribution pattern	Localized skin lesions with dermal macrophages	Systemic involvement (spleen, liver, marrow)
Iron homeostasis	Moderate iron availability, supplementation improves Th1 response	Systemic iron dysregulation and hepcidin-driven iron retention in macrophages
Oxidative stress	Early oxidative burst promotes ferroptosis while transient Nrf-2 activation protects tissue	Persistent oxidative stress with strong antioxidant response and sustained Nrf-2 activation
Immune polarization	Th1 induced M1 macrophages increase ferroptosis susceptibility	Nrf-2 induced M2 polarization supports parasite persistence and ferroptosis resistance.
Ferroptosis Susceptibility	Higher in early stages from Th1 oxidative environment but parasites evade via LPG and HO-1	Lower overall with iron trapped in macrophages and lipid peroxidation suppressed by ferritin and HO-1
Nrf-2 Role	Initially protective limiting tissue damage while sustained activation promotes parasite survival	Pathogenic by suppressing ferroptosis and immune response, supports chronic infection
Therapeutic Strategy	Early Nrf-2 activation to limit damage, followed by chronic ferroptosis induction	Nrf-2 inhibition with ferroptosis induction and iron regulation

HO-1: Heme oxygenase 1, LPG: Lipophosphoglycan.

## 4 Ferroptosis and Nrf-2 as therapeutic targets

The insightful details into ferroptosis and its complicated association with Nrf-2 have created new opportunities for developing host directed therapeutic interventions against *Leishmania* infections. Given the drawbacks of existing chemotherapeutic approaches which are marked by life threatening toxicity, emerging resistance and limited efficacy, it could be a wise approach to target host cell death pathways that could restore immune functionality and offers a new therapeutic approach.

### 4.1 Host-directed ferroptosis therapeutics

The activation of ferroptosis in infected macrophages presents a convincing host directed approach to promote intracellular parasite clearance. Ferroptosis inducing agents act primarily by disturbing the redox buffering systems that prevent lipid oxidation and target key regulatory intersections including system x_c_^−^ or GPX4. Several therapeutic agents have been developed for cancer therapy including Erastin, RSL3, Sorafenib, Sulfasalazine, FIN56 and ML210, that can be used as ferroptosis inducer and could be repurposed for leishmaniasis treatment.

Erastin is a small therapeutic drug that has been extensively studied in cancer biology and emerged as a potent inducer of ferroptosis. Erastin's mechanistic profile aligns with the redox imbalance observed in *Leishmania* infected macrophages. It works through its ability to block the system x_c_^−^ and restricts cystine import which results in depletion of intracellular glutathione and inhibits the GPX4 mediated detoxification of lipid hydroperoxides. The cell then shifts towards a ferroptotic state as lipid hydroperoxides begin to accumulate and also opposes the antioxidant defense established by Nrf-2 [44,45], during *Leishmania* infection. As mentioned earlier, Nrf-2 expands cystine-GSH network, upregulates ferritin and supports redox homeostasis, Erastin weakens this metabolic foundation on which this antioxidant me. This redox imbalance provides an opportunity to involve additional immunometabolic signals to potentiate Erastin's ferroptotic activity. In a recent study, IFN-γ is shown to limit Nrf-2 activity, downregulate SLC7A11 and facilitates Erastin's ferroptotic potential [46]. Despite lack of direct studies in *Leishmania* infections, the underlying biochemical framework and the known ability of Erastin to impair Nrf-2 directed antioxidant pathways support its consideration as a ferroptosis based therapeutic intervention against leishmaniasis.

There are several other candidate drugs. RSL3 is a small therapeutic drug that emerged as a potent inducer of ferroptosis inducer and directly inhibits GPX4. Recent studies indicate that RSL3 treatment results in increase expression of ACSL4 and PTGS2, which are known markers of ferroptosis [40]. Sorafenib, another ferroptotic inducer and multikinase inhibitor, is used for hepatic cancer therapy. It induces ferroptosis through both GPX dependent and independent mechanism. It disrupts cysteine uptake and glutathione metabolism which sensitize cells to ferroptotic death. In addition, sorafenib is also reported to weakens Nrf-2 activity, further promoting ferroptosis [47]. Sorafenib could be another potential agent for management of leishmanaisis, however, careful dose optimization is required to prevent its off target toxicity due to its broad kinase inhibition spectrum. FIN56 is another ferroptotic inducer which can function by accelerating degradation of GPX and at the same time depleting coenzyme Q10 (CoQ10), an antioxidant in cellular membrane. This depletion of CoQ10 eliminates an important defense against lipid radical propagation [48,49]. Sulfasalazine is a known anti-inflammatory drug and recently reported to inhibit system x_c_^−^, deplete cysteine uptake and GSH synthesis [50]. Similarly, atorvastatin has been shown to increase ferroptosis by depleting CoQ10 and prevent mevalonate pathway essential for GPX4 maturity which make them attractive adjuvants in host directed anti-leishmanial therapy [51]. Each of these candidates based on their ferroptotic target mechanisms could merit further study for application in leishmaniasis.

Natural compounds can also be used to induce ferroptosis in the host cells. Polyphenols such as curcumin, withaferin A, celastrol, artemisinin and piperlongumine are known compounds that promote ferroptosis by decreasing GSH, enhancing intracellular free Fe^2+^ and increasing lipid peroxidation [52,53]. Rational combinations of these phytochemicals with conventional or ferroptosis inducing drugs may yield synergistic outcomes.

An additional therapeutic approach centers on modifying iron homeostasis and could use intracellular free iron increasing agents like ferric ammonium citrate or low dose iron sucrose to increase ferroptotic sensitivity [54]. A combined strategy including controlled increase in intracellular free iron and inhibition of GPX4 may offer a promising strategy against leishmaniasis, however, precise iron regulation is necessary since excessive iron supplementation may exacerbate tissue pathological changes and increase chances of secondary infections.

Induction of ferroptosis inducing agents can be used in combination with established antileishmanial drugs. Studies indicated that amphotericin B (AmB) and miltefosine (Milt) exert oxidative stress on parasites but fail to completely eradicate due to antioxidant compensation [55]. These known drugs can be combined with ferroptosis inducers which could amplify lipid peroxidation beyond the *Leishmania's* tolerance threshold. Given the mechanistic overlap in redox and metabolic pathways, ferroptosis inducers may increase the anti-leishmanial efficacy of known anti-leishmanial drugs. It is known that AmB enhances cellular iron uptake and promotes ROS production which creates a redox environment that could facilitate macrophages to GPX inhibition by RSL3 induced ferroptosis. Likewise, Milt, which targets mitochondrial metabolism and inhibits intracellular glutathione, could be used in combination with erastin, which decreases system xc-mediated cysteine import, further depleting GSH levels. Although experimental confirmation of these combination approaches has not yet been established in *Leishmania* infection, the overlapping mechanisms present a strong scientific justification for evaluating these combinations.

### 4.2 Host-directed Nrf-2 therapeutics

The Nrf-2 signaling pathway is essential for limiting oxidative injury in the host cells, however, this host protective response promotes intracellular parasite survival through suppression of ferroptosis and reduction of immune activation. Emerging evidence indicates that Nrf-2 signaling exhibits different outcomes across different phenotypes of leishmaniasis. In CL, excessive inflammation and ROS generation is observed which contribute to tissue pathology and a brief Nrf-2 activation may be helpful to balance oxidative stress while protecting macrophages anti-parasitic potential. However, prolonged Nrf-2 activation shifts macrophages toward an immunoregulatory state that promotes disease progression. In VL, Nrf-2 activation supports parasite survival by increasing antioxidant genes such as GPX4, HO-1 and SLC7A11 which helps the parasites in limiting ferroptotic damage. Thus, while early Nrf-2 activation may help control oxidative stress in CL, prolonged activation support parasite survival in both CL and VL. Therapeutic inhibition of Nrf-2 or its downstream targets could enhance ferroptosis and accelerate parasite clearance.

Brusatol is one of the potent inhibitors of Nrf-2 and reported to suppress this antioxidant pathway across different experimental settings [56]. It accelerates the ubiquitin derived degradation of Nrf-2 which in turn decreases the expression of downstream antioxidant genes and disrupts the cellular defense programs that protects against oxidative stress. Brusatol induced inhibition of Nrf-2 is shown to increase susceptibility in multiple cancer cell lines and xenograft models to chemotherapeutic agents, proving its functional specificity and biological potency. Therefore, brusatol could serve as a promising candidate for the therapeutic intervention of VL, either alone or in combination with ferroptosis inducing agents. Similarly, ML385 is another selective inhibitor that interrupts Nrf-2 binding to antioxidant response elements (AREs) and leads to ferroptotic death of infected macrophages. Several studies in acute myeloid leukemia, epithelial cell injury, T-cell leukemia and tamoxifen resistant breast cancer report that ML385 suppresses Nrf-2 mediated transcription, decreases GPX4 and HO-1 activities, and increases intracellular iron and ROS production. It also significantly increases the cytotoxic potential of other therapeutic drugs such as venetoclax or RSL3. ML385 increases ferroptotic death by promoting lipid peroxidation, even in settings where Nrf-2 is pathologically upregulated [[57], [58], [59]]. These outcomes highlight ML385 as a promising anti-leishmanial therapeutic candidate where it could induce ferroptosis death in infected macrophages and weaken the antioxidant shield of *Leishmania* parasites.

As mentioned earlier, HO-1 is a key downstream effector of Nrf-2 and maintains an important position in the iron-redox interplay within *Leishmania* infected macrophages. In a study, Nrf-2-HO-1 blockage using Nrf-2 inhibitor trigonelline hydrochloride restores oxidative vulnerability in *L. donovani* infected macrophages which results in increase lipid peroxidation, rupture of mitochondrial membranes and parasite killing [43]. Zinc protoporphyrin IX (ZnPP) is another known HO-1 inhibitor and has been used in models of malaria [60], cancer [61] and liver fibrosis [62], where it shifts cells toward a more pro-oxidant state and counter the oxidative stress neutralizing and cytoprotective functions of HO-1. These studies highlight the therapeutic potential of HO-1, especially the study of malaria parasites which showed that HO-1 inhibition with ZnPP inhibited HO-1 mediated iron overload, reduced lipid peroxidation and significant reduction in parasite accumulation and proliferation. These findings could be extrapolated to leishmaniasis where HO-1 blockage by ZnPP may weaken the parasite favorable environment inside the infected macrophages and shift the redox iron balance against the parasites. Therefore, ZnPP and related HO-1 inhibitors could be promising candidates for host mediated anti-leishmanial therapeutics.

On the other hand, recent studies highlight the therapeutic relevance of Nrf-2 activators in managing dermatological and inflammatory skin conditions, supporting their potential in early CL, a stage marked by oxidative stress induced tissue pathology. Several compounds including hydroxytyrosol, sulforaphane, dimethyl fumarate (DMF) and pentalinonsterol (PEN) have shown the capacity to limit ROS derived injury, promote wound repair and promote anti-inflammatory macrophages polarization across various experimental conditions of radiation damage, obesity induced cutaneous dysfunction and other inflammatory conditions [[63], [64], [65], [66], [67]] In particular, PEN is a novel Nrf-2-activating drug which was recently reported in the context of early CL. PEN stabilized the redox balance, limited lipid peroxidation, and protects host tissue from excessive oxidative damage by promoting nuclear translocation of Nrf-2 and enhancing the expression of antioxidant and cytoprotective enzymes PEN's dual ability to regulate inflammation and supporting antioxidant mechanisms positions PEN as a promising host directed therapeutics for early CL, aimed at preserving tissue integrity and supporting effective immune responses against *Leishmania* parasites. An overview of promising compounds modulating ferroptosis and Nrf-2 pathways for leishmaniasis treatment is presented in Table 3, including their mechanisms of action and therapeutic relevance in leishmaniasis.

**Table 3:** Potential drug candidates targeting ferroptosis and Nrf-2 signaling in leishmaniasis.

Drug Candidates	Functional Classification	Mechanism of Action	Therapeutic Relevance in Leishmaniasis	Comments	References
Erastin	Ferroptosis Inducer	Disrupts X_c_^−^/GSH/GPX4 axis and promotes lipid peroxidation	Inhibits Nrf-2 induced antioxidant response	Repurposed from cancer	[44,45]
RSL3	Ferroptosis Inducer	Directly inhibits GPX4, upregulates ACSL4 and PTGS2	Enhances lipid peroxidation in VL and may synergize with AmB	Increases ferroptosis markers and redox imbalance.	[40]
Sorafenib	Ferroptosis Inducer	Inhibits cysteine uptake, GSH metabolism and Nrf-2 activity	Overcomes antioxidant shields in CL/VL	Multikinase inhibitor used in liver cancer	[47]
FIN56	Ferroptosis Inducer	Accelerates GPX degradation and depletes CoQ10	Potential to overcome parasite antioxidant mechanisms	Repurposed from cancer	[48]
Brusatol	Nrf-2 Inhibitor	Promotes Nrf-2 ubiquitin degradation and lowers antioxidants (HO-1, GPX4)	Disrupts antioxidant environment and enhances ferroptosis to reduce parasite survival	Effective in cancer models	[56]
ML385	Nrf2 Inhibitor	Inhibits Nrf-2 binding, reduces GPX4/HO-1, increases ROS and iron	Promotes ferroptosis in infected macrophages and may synergize with RSL3	For leukemia and breast cancer	[[57], [58], [59]]
Zinc Protoporphyrin IX (ZnPP)	HO-1 Inhibitor (Nrf-2 Downstream)	Promotes pro-oxidant and lowers iron defense	Weakens parasite-supportive environment in VL and limits proliferation similar to malaria models	Malaria and cancer use, potential for *Leishmania*	[60,62]
Curcumin	Ferroptosis Inducer (Natural)	Reduces GSH and elevates Fe^2+^ with lipid peroxidation	Adjuvant in combinations enhancing oxidative stress beyond parasite tolerance	Synergistic polyphenol with conventional drugs	[53]
Pentalinonsterol	Nrf-2 Inducer	Stabilizes Nrf-2 and upregulates antioxidant genes while reducing excessive ROS	Supports tissue protection and wound healing in early CL	Natural compound with anti-inflammatory effect	[67]

ACSL4: Acyl-CoA synthetase long chain family member 4, AmB: Amphotericin B, ARE: Antioxidant response element, CL: Cutaneous leishmaniasis, CoQ10: Coenzyme Q10, FCC: Ferritin chaperone cleavage, GPX4: Glutathione peroxidase 4, GSH: Glutathione, HO-1: Heme oxygenase 1, Nrf-2: Nuclear factor erythroid 2 related factor 2, PTGS2: Prostaglandin-endoperoxide synthase 2, ROS: Reactive oxygen species, VL: Visceral leishmaniasis, ZnPP: Zinc protoporphyrin IX.

### 4.3 Combination therapy in ferroptosis regulation

Ferroptosis is regulated through multiple pathways, making combination therapeutic strategies that regulate iron metabolism and lipid peroxidation potentially advantageous. Iron chelators including deferoxamine, though effective in limiting parasite replication, can unexpectedly suppress ferroptosis when used in excess. Therefore, pairing moderate iron chelation with ferroptosis inducers may enhance synergistic benefits by restricting parasite access to iron while maintaining sufficient active iron for lipid peroxidation. Several novel iron chelators including Dp44mT and deferiprone analogs are under investigations [27].

**Fig. 3:**
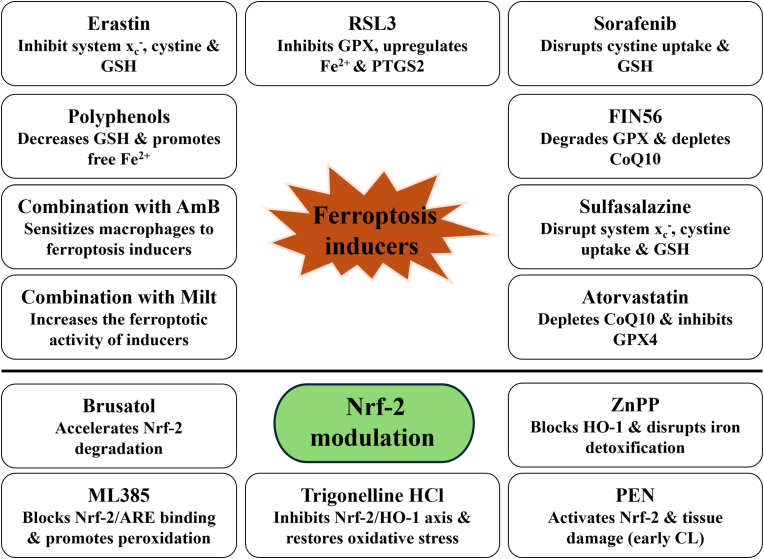
The figure shows selected ferroptosis inducers and Nrf-2 modulating drugs that could be exploited for leishmaniasis therapy. Inhibition of antioxidant mechanisms or enhancement of lipid peroxidation promotes ferroptosis, leading to parasite clearance. Conversely, activation of Nrf-2 driven cytoprotective responses during early CL restores redox balance, prevents lipid peroxidation and limits collateral tissue damage.

In addition, ferroptotic responses can also be potentiated by targeting lipid metabolism. ACSL4 inhibitors could prevent the development of phospholipids that are vulnerable to oxidation while promotion of ACSL4 expression could enhance ferroptosis [68]. Dietary modification such as using omega-3 fatty acids which increase PUFA incorporation into host cell membranes may further trigger infected cells to ferroptotic death [69]. Some of the important ferroptosis and Nrf-2 modulating agents that could be used in the leishmaniasis research is shown in Fig. 3.

Although cutaneous and visceral leishmaniasis are caused by distinct *Leishmania* species, it remains unclear whether ferroptosis and Nrf-2 related pathways are engaged through conserved or species-specific mechanisms. Studies in human CL skin lesions report elevated Nrf-2/HO-1 signaling, increased lipid peroxidation markers such as malondialdehyde and altered antioxidant enzyme activities, indicating substantial redox and lipid oxidative stress during infection [70]. While these findings are consistent with a ferroptosis permissive environment, direct mechanistic evidence linking these alterations to regulated ferroptotic cell death across different *Leishmania* species remains limited, highlighting the need for comparative cross-species investigations.

### 4.4 Nanotechnology-driven ferroptosis therapeutics

Nanotechnology based drug delivery systems can not only be used to optimize efficacy and reduce systemic toxicity but also help in targeting ferroptosis and Nrf-2 modulators specifically to *Leishmania* infected macrophages. Polymeric, iron or lipid based nanoparticles (NPs) can be engineered to actively target macrophages through attachment of macrophage specific ligands such as mannose, folate or dextran sulfate and these nanoparticles will be actively uptaken by macrophages via receptor mediated endocytosis [71]. These nanoparticles will then release the drug inside the macrophages specifically within the parasitophorous vacuoles, where *Leishmania* parasites are present and ensure localized ferroptotic induction.

Recent studies highlight that iron based NPs serve as effective inducers of ferroptosis and present a promising therapeutic strategy against intracellular *Leishmania* parasites. Plain iron NPs, without any drug payload, possess intrinsic ferroptotic activity by releasing active bioavailable Fe^2+^ and catalyzing Fenton chemistry which drives lipid peroxidation. Several studies reported that iron NPs engineered with gallic acid/polyacrylic acid coated formulations or PEGylated superparamagnetic iron oxide system loaded with erastin (a ferroptosis inducer), resulted in a significant increase in ROS, elevated intracellular active iron pool and decreased GPX4 activity with accumulation of toxic lipid hydroperoxides which ultimately triggered ferroptotic death in cancer models. Notably, a synergistic effect in promoting ferroptosis was observed by combining erastin with iron oxide NPs due to simultaneous inhibition of system x_c_^−^ and iron derived amplification of ROS [72,73]. These mechanistic insights are translatable to leishmaniasis, where *Leishmania* parasites are completely dependent on host iron homeostasis and antioxidant defense systems to evade oxidative killing mechanism. Therefore, the application of nanotechnology, especially iron based NPs could be employed as a potential new candidate to induce ferroptosis in *Leishmania* infected macrophages and could establish an innovative frontier in the search for effective anti-leishmanial interventions.

### 4.5 Ferroptosis/Nrf-2 in immunotherapy and vaccines

In recent years, the relationship between host immunity and ferroptosis in parasitic diseases is being increasingly studied at the molecular level. The accumulation of lipid peroxidation products such as malondialdehyde (MDA) and 4-hydroxynonenal (4-HNE) not only act as biomarkers for ferroptosis but also function as a signaling molecules for inflammation, contributing to immune modulation. These products can activate pattern recognition receptors and stimulate cytokine release which links innate immune activation to ferroptotic cell death [74,75]. These findings suggest that ferroptosis driven oxidative stress interacts with immune pathways and offers potential insight for designing vaccine or immunotherapeutic strategies. Therefore, modulation of ferroptosis and Nrf-2 pathway may have important associations for immunotherapy and vaccine development strategies broadly. The release of oxidized lipid antigens and damage associated molecular pattern in response to ferroptosis promote dendritic cell maturation and enhance antigen presentation which provide an immunological advantage for vaccine development [76]. In *Leishmania* infected macrophages, controlled induction of ferroptosis may theoretically enhance immune activation while preventing excessive tissue inflammation. On the other hand, inhibition of Nrf-2 during vaccine administration might potentiate oxidative pathways that drive Th1 polarization which is essential for protective response against leishmaniasis. These theoretical concepts may provide a conceptual foundation for future efforts in translational science.

## 5 Translational challenges and future directions

Research increasingly supports the idea that modulating ferroptosis and Nrf-2 activity could benefit host-directed treatment of leishmaniasis; however, critical steps in translation remain unresolved. Critical limitations relate to pharmacokinetic and pharmacodynamic factors, especially the question of whether ferroptotic agents can reach sufficient levels in infected organs and selectively penetrate parasitized macrophage populations. Systemic ferroptosis induction or extended Nrf-2 suppression may presents toxicity problems, as excessive lipid peroxidation or impaired antioxidant defenses can inflict tissue damage, promote inflammation and harm uninfected cells. Furthermore, the majority of ferroptotic agents have been assessed in *in* *vitro* or in animal models, and the data on efficacy in humans safety and bioavailability is lacking.

To address these challenges, future research should prioritize targeted delivery strategies to enhance tissue specificity and minimize systemic toxicity. Complementary *in vivo* studies and comparative analyses across CL and VL models are also needed to clarify the context dependent role of ferroptosis and Nrf-2. Together, these advances will help translate mechanistic findings into host directed therapeutic strategies that safely utilize ferroptotic pathways and Nrf-2 linked antioxidant responses.

## 6 Conclusions

The initial studies linking ferroptosis and Nrf-2 signaling to the pathophysiology of *Leishmania* infection are encouraging, however, the application of these findings from mechanistic knowledge to therapeutic development is still at an early stage. The anti-leishmanial therapeutic strategies focused on ferroptosis and Nrf-2 are limited by uncertainties in underlying mechanisms, significant translational challenges and unresolved safety considerations. To address these gaps, a comprehensive approach integrating redox biology, parasitology, immunometabolism and drug delivery science is essential for understating host parasite relationships and development of host directed therapeutics designed to exploit ferroptotic and Nrf-2 mechanisms for effective management of leishmaniasis.

## Declaration of generative AI and AI-assisted technologies in the writing process

During the preparation of this work the author(s) used Microsoft co-pilot in order to improve language and readability only. After using this tool/service, the author(s) reviewed and edited the content as needed and take(s) full responsibility for the content of the publication.

## CRediT authorship contribution statement

**M. Junaid Dar:** Conceptualization, Writing – original draft, Writing – review & editing. **Ryan H. Huston:** Writing – review & editing. **Nandithadas Purayil:** Writing – original draft, Writing – review & editing. **Ashley I. Cronin:** Writing – original draft. **Sonia Ashfaq Gondal:** Writing – original draft, Writing – review & editing. **Hira L. Nakhasi:** Supervision, Writing – review & editing. **Reginaldo Brito:** Writing – original draft. **Sreenivas Gannavaram:** Writing – review & editing. **Abhay R. Satoskar:** Conceptualization, Resources, Supervision.

## Declaration of competing interest

This article reflects the views of the authors and should not be construed to represent FDA's views or policies. The remaining authors declare no conflict of interest.

## Data Availability

No data was used for the research described in the article.
